# Rationale and study design of an early care, therapeutic education, and psychological intervention program for the management of post-intensive care syndrome and chronic pain after COVID-19 infection (PAIN-COVID): study protocol for a randomized controlled trial

**DOI:** 10.1186/s13063-021-05463-7

**Published:** 2021-07-24

**Authors:** Antonio Ojeda, Andrea Calvo, Tomas Cuñat, Ricard Mellado Artigas, Oscar Comino-Trinidad, Jorge Aliaga, Marilyn Arias, Maribel Ahuir, Carlos Ferrando, Christian Dürsteler

**Affiliations:** 1 Pain Medicine Section, Department of Anaesthesiology, Critical Care and Pain Treatment, Hospital Clínic de Barcelona, Barcelona, Spain; 2 Surgical Intensive Care Unit, Department of Anesthesiology, Critical Care and Pain Treatment, Hospital Clínic, Institut d’investigació August Pi i Sunyer, 08036 Barcelona, Spain; 3 Department of Anaesthesiology, Critical Care and Pain Treatment, Hospital Clínic de Barcelona, Barcelona, Spain; 4 Department of clinical Psychology, Clinical Neuroscience Institute, Hospital Clínic de Barcelona, Barcelona, Spain; 5 CIBER de Enfermedades Respiratorias, Instituto de Salud Carlos III, Madrid, Spain

**Keywords:** COVID-19, Randomized controlled trial, Protocol, Post ICU syndrome, Chronic pain, Critical illness

## Abstract

**Background:**

Critically ill patients with COVID-19 are an especially susceptible population to develop post-intensive care syndrome (PICS) due to acute respiratory distress syndrome (ARDS). Patients can suffer acute severe pain and may have long-term mental, cognitive, and functional health deterioration after discharge. However, few controlled trials are evaluating interventions for the prevention and treatment of PICS. The study hypothesis is that a specific care program based on early therapeutic education and psychological intervention improves the quality of life of patients at risk of developing PICS and chronic pain after COVID-19. The primary objective is to determine whether the program is superior to standard-of-care on health-related quality of life at 6 months after hospital discharge. The secondary objectives are to determine whether the intervention is superior to standard-of-care on health-related quality of life, incidence of chronic pain and degree of functional limitation, incidence of anxiety, depression, and post-traumatic stress syndrome at 3 and 6 months after hospital discharge.

**Methods:**

The PAINCOVID trial is a unicentric randomized, controlled, patient-blinded superiority trial with two parallel groups. The primary endpoint is the health-related quality of life at 6 months after hospital discharge, and randomization will be performed with a 1:1 allocation ratio. This paper details the methodology and statistical analysis plan of the trial and was submitted before outcome data were available.

The estimated sample size is 84 patients, 42 for each arm. Assuming a lost to follow-up rate of 20%, a sample size of 102 patients is necessary (51 for each arm).

**Discussion:**

This is the first randomized clinical trial assessing the effectiveness of an early care therapeutic education, and psychological intervention program for the management of PICS and chronic pain after COVID-19. The intervention will serve as proof of the need to implement early care programs at an early stage, having an incalculable impact given the current scenario of the pandemic.

**Trial registration:**

This study is being conducted in accordance with the tenets of the Helsinki Declaration and has been approved by the authors’ institutional review board *Comité Ético de Investigación Clínica del Hospital Clínic de Barcelona* (approval number: HCB/2020/0549) and was registered on May 9, 2020, at clinicaltrials.gov (NCT04394169).

**Supplementary Information:**

The online version contains supplementary material available at 10.1186/s13063-021-05463-7.

## Introduction

Since the initial outbreak of COVID-19 in December 2019, there have been more than 14.3 million cases of infection with the SARS-CoV-2 virus reported worldwide [1]. This has led to a large number of hospital admissions testing the capability of many healthcare systems. Among hospitalized patients, 10–20% are admitted to the intensive care unit (ICU), and more than 70% of those require invasive mechanical ventilation with an overall mortality over 30% [2–4].

Critical care survival has been reported as 16–37%, and this will result in an unimaginable size of a cohort of critical care survivors given the number of global infections; these patients can experience a significant worsening of their health status and deterioration of their quality of life [5]. In 2012, the Society of Critical Care defined a new term, the post-intensive care syndrome (PICS), as the worsening of the physical, mental or cognitive patient’s status after a critical illness that is maintained beyond hospitalization. Consequently, health-related quality of life (HRQoL) and post-intensive care syndrome (PICS) is becoming the focus of intensive care medicine rather than survival rate alone [6, 7]. As psychological dysfunction can persist for years after ICU discharge, its management is becoming an important strategy to improve quality of life together with early detection of posttraumatic stress disorder and anxiety and depression [8]. Moreover, there is evidence that patients who survive a critical illness have a high prevalence of moderate to extreme chronic pain being an essential factor affecting their ability to return to work and to restore quality of life for up to 5 years following discharge [9]. However, few controlled trials have evaluated interventions for the prevention and treatment of PICS [10].

Patients with COVID-19 are an especially susceptible population to develop PICS due to acute respiratory distress syndrome (ARDS). Patients can suffer acute severe pain and may have long-term deterioration in their mental, cognitive, and functional health after discharge. However, pain is a conscious experience by definition; thus, sedated patients can suffer a high nociceptive input inadvertently [5]. However, there are recent publications describing nervous system involvement after infection with SARS-CoV-2 [11, 12]. Thus, chronic pain could potentially occur as a complication or sequel of COVID-19. However, as far as we know, there are no studies related to chronic pain care after a critical illness, specifically in patients with COVID-19 [13].

We hypothesize that a specific care program based on early therapeutic education and a psychological intervention improves the quality of life of patients at risk of developing PICS and chronic pain after COVID-19.

### Primary outcome

The primary objective is to determine if a specific care program based on early therapeutic education and a psychological intervention improves the health-related quality of life (HRQoL) compared to standard care at 6 months after hospital discharge.

### Secondary outcomes

The secondary objectives are to determine if the intervention is superior to standard-of-care, by evaluating:

- The HRQoL at three months after hospital discharge.
- The incidence of chronic pain and the degree of functional limitation at 3 and 6 months after hospital discharge.
- The incidence of anxiety and depression at 3 and 6 months after hospital discharge.
- The incidence of post-traumatic stress syndrome at 3 and 6 months after hospital discharge.

## Methods

### Study design

**Fig. 1:**
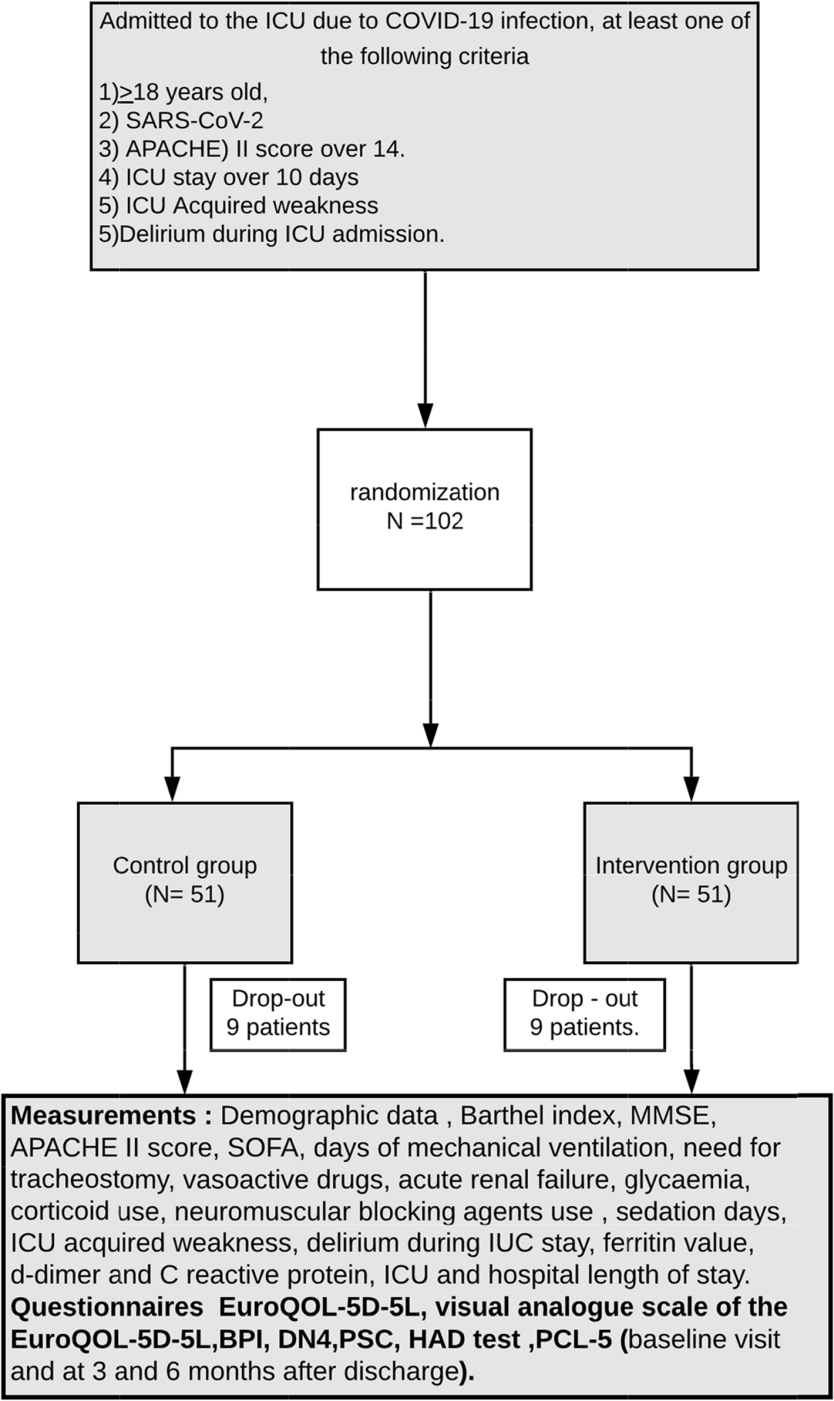
Summary of patient flow diagram

The PAIN-COVID is a comparative, prospective, single-center randomized controlled trial that will include 102 patients (Fig. 1). The trial has been designed in accordance with the fundamental principles established in the Declaration of Helsinki, the Convention of the European Council relating to human rights and biomedicine, and the Universal Declaration of UNESCO on the human genome and human rights, and with the requirements established by Spanish legislation in the field of biomedical research, the protection of personal data, and bioethics, registered on May 9, 2020, at http://www.clinicaltrials.gov with identification No. (NCT04394169). Approval of the final protocol by the *Comité Ético de Investigación Clínica del Hospital Clínic de Barcelona*—approval number: HCB/2020/0549, Chairperson: Prof Joaquin Fores Viñeta, on May 14, 2020.

**Fig. 2:**
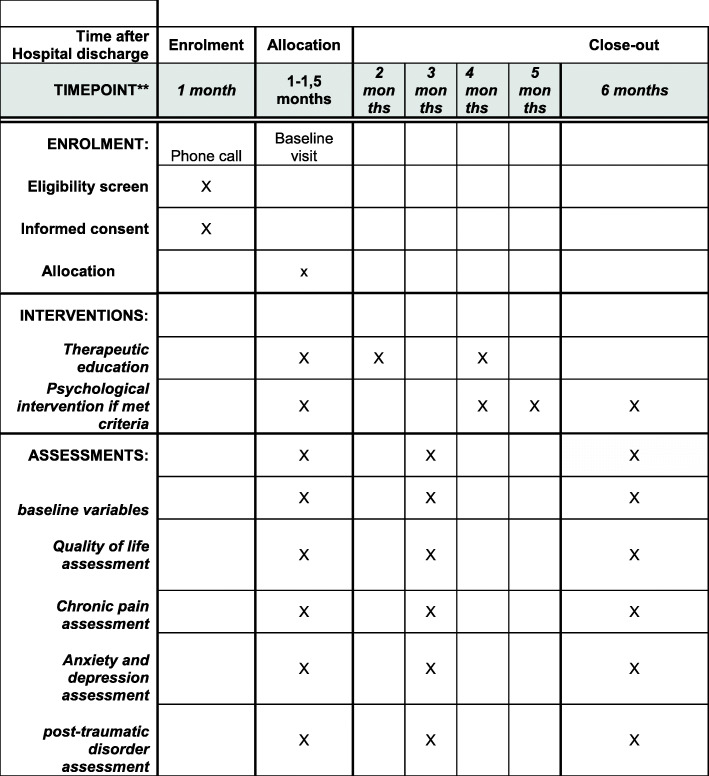
Schedule of enrolment interventions and assessment, SPIRIT 2013

This study followed the “Standard Protocol Items: Recommendations for Interventional Trials.” The SPIRIT 2013 Statement provides evidence-based recommendations for the minimum content of a clinical trials protocol (Fig. 2).

### Study population

Adult patients will be enrolled if they fulfil at least one of the following Inclusion criteria: (1) had SARS-CoV-2 infection, confirmed with a respiratory tract sample using PCR-based tests, (2) had an Acute Physiology And Chronic Health Evaluation (APACHE II) score over 14, (3) ICU stay over 10 days, (4) acquired weakness in ICU [14] (Supplement, Definition D1), (5) delirium during ICU [14] (Supplement Definition, D2), and 6) acceptance to participate in the study by signing the informed consent form.

The exclusion criteria are as follows: (1) patients with non-confirmed SARS-CoV-2 infection according to WHO guidance [15], (2) central ervous system degenerative diseases or terminal illness (Supplement, Definition D3), (3) terminal illness (Supplement, Definition D4) [16], (4) insufficient understanding of the Spanish language, (5) patients with whom it would be difficult to complete follow-up, and (6) not willing to sign the informed consent form.

### Methods of randomization and bias minimization

Once the informed consent has been obtained, the patient will be assigned 1:1 by the investigator to either the control or intervention group according to the allocation sequence generated by the randomization program. Only the researchers who signed the informed consent had access to this list which was concealed from the principal investigator.

The data for the screening will be obtained from the clinical records program of the Hospital Clínic de Barcelona, where the data of patients who required admission to the ICU, as well as days of admission, will be reviewed by the members of the research team in order to verify whether they are eligible or not.

Screening for patients will be done monthly. Enrolment is expected to be completed within 3 to 6 months. The baseline visit will take place between 4 to 6 weeks after hospital discharge. Follow-up visits will take place at 3 and 6 months after discharge (Fig. 2.)

Patients will be encouraged to remain in the study during each interview by giving them feedback about the importance of their collaboration. Participants who decide to drop out during follow-up will be asked about the reason for it, and all their answers will be recorded.

The database will be paper-based and electronic data entry will also be used using FileMaker. Only three research team members will obtain access to this program. case report forms (CRF) will be kept in the hospital files and all data will be available on the web after study finalization.

### Blinding

This is a single-blind study. Visits will be carried out by an investigator with adequate training in questionnaire administration. This investigator will not participate in the intervention or the evaluation of the results. The intervention will be performed by two researchers (a pain physician and a psychologist). These researchers will not participate in the questionnaire and baseline data collection nor in the data analysis. Researchers who analyze the results will not participate in the questionnaire and baseline data collection or program intervention.

#### General procedures

The study subjects will be assigned to one of two arms, and the intervention program will be compared to the standard-of-care clinical practice. The baseline visit will take place 4 to 6 weeks after hospital discharge, and two follow-up visits will take place 3 and 6 months after.

The intervention program will consist of an early care therapeutic education on prevention and management of PICS and chronic pain during the three scheduled medical visits within the first 6 months after hospital discharge and psychological treatment for patients at risk of emotional distress.

### Recruitment and participant timeline

Patients who are eligible for the study will be contacted approximately 1 month after discharge from the hospital, they will be informed about the study, and they will be asked to participate. Those who accept to participate will have to complete a baseline visit the following week. Informed consent will be obtained by one of the investigators (Supplement, Figure F2).

#### Baseline visit

The baseline visit will take place between 4 to 6 weeks after hospital discharge. Information regarding the study will be given to the patient, and informed consent will be obtained. After that, the patient will be randomized. During this first visit, demographic data, medical history, and ICU and hospitalization variables will be collected from all the included patients, regardless of their randomization arm. All patients will complete a series of questionnaires to evaluate their quality of life and the presence of anxiety, depression, or post-traumatic stress disorder. The presence of pain and its influence on the patients’ lifes will also be evaluated.

Intervention group

The intervention consists of a program that includes early patient care, therapeutic education, and psychological intervention. It will be implemented across three medical visits scheduled as follows:

- Visit 1 Intervention group, 4 to 6 weeks after hospital discharge.
- Visit 2 Intervention group, 8 weeks after hospital discharge.
- Visit 3 Intervention group, 18 weeks after hospital discharge.

Components:

- Interview and physical examination.
- Therapeutic education about the PICS, orally and in writing with specific documents delivered at the end of the visit, i.e., a PICS fact-sheet developed by the investigators and a rehabilitation manual recommended by the Follow-up and Rehabilitation Committee of the Argentine Society of Intensive Care, SATI [14].
- Therapeutic education about pain (if the patient reports pain) which includes an explanation of pain neurophysiology, the rational use of drugs prescribed by other specialists, information about how to manage daily life activities, and the importance of pre-emptive pain management for proper rehabilitation.

A psychological intervention will be conducted if the following criterium is met: a score higher than 8 on the hospital anxiety and depression (HAD) test depression subscale (supplement, questionnaire Q1) [17]. The intervention protocol will consist of 7 weekly sessions lasting one and a half hours each (supplement, Table 1). The intervention in depression is based on Rehm’s model of self-control. Psychological interventions may cause adverse events resulting in worsening of patients’ clinical course (overdose, self-harm, and self-harm attempts). Therefore, the investigators will monitor any related symptom, report it as an adverse event, and refer the patient for treatment by a specialist unit.

#### Control group

Standard-of-care is as follows: patient follow-up will be carried out by their referring physicians (primary care physicians or specialists), who will not be involved in the study. After the baseline visit, the second and third visits will be phone call visits at 3 and 6 months after hospital discharge.

#### Outcome measurements

Demographic data will be collected at baseline visit, including age, gender, body mass index, smoking habits, socioeconomic level, work status, and marital status. Barthel index and medical history will also be recorded, especially psychiatric disorders, chronic pain, opioid usage, and previous ICU admission (Supplement, Table 2).

Data regarding ICU and hospital admission will be also collected: Acute Physiology and Chronic Health disease Classification System (APACHE II) and Sequential Organ Failure Assessment Score (SOFA) severity scores, days under invasive or non-invasive mechanical ventilation, presence of sepsis [18] (Supplement Definition, D5), need for tracheostomy, use of vasoactive drugs, acute kidney injury (Supplement Definition, D6) and need for renal replacement therapy, stress hyperglycaemia and hypoglycaemia (Supplement Definition, D6, D7), corticoid use, use of neuromuscular blocking agents, days under sedation, ICU acquired weakness, delirium presence, maximum value of ferritin, d-dimer and C reactive protein, and ICU and hospital length of stay. The Mini-Mental State Exam (MMSE) test, which is a widely used test of cognitive function among the elderly that assesses orientation, attention, memory, language, and visual-spatial skills, will be evaluated before answering the questionnaires [19].

The impact of the intervention program on health-related quality of life reported by the patient will be assessed through the European quality of life 5 dimensions/5 levels (supplement, questionnaire Q2) [20]. The questionnaire assesses the quality of life of study participants according to 5 domains: mobility, self-care, usual activities, pain/discomfort, and anxiety/depression, each scored according to a scale of 1 (no problems) to 5 (extreme problems) and generating a 5-digit code corresponding to the quality of life. The visual analog scale of the same test will also be assessed (from 0—worst imaginable health—to 100—best imaginable health). The questionnaire provides a simple descriptive profile of a respondent’s health status. Quality of life will be assessed at Baseline Visit and at 3 and 6 months after discharge.

Pain (presence and intensity) will be assessed by the Brief Pain Inventory (BPI) questionnaire (supplement, questionnaire Q3) [21] at the baseline visit and at 3 and 6 months after discharge. This questionnaire is a multidimensional questionnaire that evaluates pain intensity in the last 24 hours (worst, lowest, average) and current (right now). It also assesses the impact of pain on daily activities (general activity, encouragement, work, social interaction, sleep, enjoyment of life and the ability to walk). The questions are rated on a scale from 0 to 10, with 10 being the worst possible value. Subsequently, the average intensity score (BPI intensity score) and average interference score (BPI interference score) is calculated. Following IMMPACT recommendations, a clinically significant pain will be recorded if the mean intensity score (BPI intensity score) is greater than or equal to 3 [22].

If BPI is positive for pain, pain catastrophizing will be assessed by the Pain Catastrophizing Scale (PCS) (supplement, questionnaire Q4) [23] and patients will also complete the *Douleur Neuropathique en 4 Questions* (DN4) (supplement, questionnaire Q5) to screen for neuropathic pain [24]. The PCS consists of 13 questions that explore the frequency of thoughts and feelings that the interviewees have in the presence of current or anticipated pain, which are grouped into three scoring subscales (magnification, rumination and defencelessness). Each question is rated on a 5-point scale (0: not at all; 4: all the time). The maximum total score is 52 points. A score greater or equal than 30 will be considered as a clinically relevant level of catastrophizing.

The impact of the intervention program on the incidence of anxiety or depression will be assessed by the Hospital Anxiety and Depression test (HAD) (supplement, questionnaire Q1) [17], consisting of 14 questions, with two subscales, one for anxiety and the other for depression, with seven items each and a maximum score of 21 for each subscale. The cut-off points are as follows: 0 to 7 imply the absence of clinically relevant anxiety and depression, 8 to 10 imply de presence of symptoms that require consideration, and 11 to 21 it report the presence of relevant symptoms, with a very probable diagnosis of anxiety or depression. According to Bjelland’s review, cut-off points equal or greater that 8 will be used as abnormal anxiety or depression’s values. This test will be performed at the baseline visit and at 3 and 6 months [25].

Finally, the incidence of post-traumatic stress disorder (PTSD) [26] will be evaluated with the post-traumatic stress disorder checklist questionnaire (PCL-5) [27]. It contains 20 questions that correspond to the DSM-V criteria. Participants will rate their symptoms on a scale of 0 (not at all), 1 (slightly), 2 (moderately), 3 (quite) to 4 (extremely), with a score ranging from 0 to 80. The overall severity of the symptoms can be assessed adding up the scores of each question (interval 0–80). The severity of each symptom can also be evaluated adding up the scores of the questions. DSM-5 symptom cluster severity scores can be obtained by adding up the scores for the items within a given cluster, i.e., cluster B (items 1–5), cluster C (items 6–7), cluster D (items 8–14), and cluster E (items 15–20). A provisional PTSD diagnosis can be made by treating each item rated 2 (“moderately”) or higher as a symptom endorsed, then following the DSM-5 diagnostic rule which requires at least: 1 B item (questions 1–5), 1 C item (questions 6–7), 2 D items (questions 8–14), and 2 E items (questions 15–20) (supplement, questionnaire Q6).

For this analysis, questionnaire licensing was obtained. The validated version in Spanish was used for each of them, except for PCL-5 which, being a new questionnaire, is not yet validated in Spanish, but it has the advantage of screening PTSD according to the DSM-V criteria. The questionnaires are shown in the supplement.

### Statistical methods

#### Sample size

To calculate the sample size of the PAIN COVID clinical trial with an assumed average of 50 points on the visual analog scale of the EuroQOL-5D-5L for the control group, and a clinically relevant difference between the groups of 20%, for distribution of a tail with a type I error of 0.05 and a power of 80%, the sample size has been calculated as 84 patients, 42 for each arm. Assuming a loss to follow-up of 20%, the sample size needed is 102 patients (51 in each group).

### Data analysis

Qualitative variables will be presented as proportions, while for quantitative variables, the mean (standard deviation) or median (interquartile range), after checking for normality using the Shapiro-Wilk test, will be used. To compare variables across groups, Student *t* tests or Mann-Whitney *U* tests for continuous data and chi-square tests or exact tests for categorical variables will be carried out. Before parametric hypothesis testing, equality of variances will be studied using the Levene’s test, and if assumptions are not met, contrasts will be performed with the Welch’s test. An intention-to-treat approach will be followed. Two-tailed *P* values will be presented and a significance level of 0.05 will be used.

For the secondary outcomes, adjustment with the Benjamini-Hochberg procedure will be carried out.

A sub-analysis of the effect of treatment on compliers will be performed for the main outcome. Compliers are defined as those subjects that effectively receive the treatment they are allocated to. For the present study, compliers will be defined as individuals that, having been randomized to the intervention, complete at least two out of three medical visits and at least five out of seven psychological interventions. For the statistical analysis, instrumental variable analysis will be carried out. A two-sided probability (*p*) value of less than 0.05 will be considered to indicate statistical significance. The statistical analysis was performed using R (https://www.rstudio.com/) statistical software.

### Dissemination policy

Investigators will communicate the trial results to participants, healthcare professionals, and the general public by posting them in results databases. The purpose of the data collection is specific to achieving the objectives defined in the project. The data collected during the study will be included in the investigators file master owned by the center.

The treatment and communication of personal data of all participants will be in compliance with the Regulation EU 2016/679 of the European Parliament and of the Council of April 27, 2016, on the protection of natural persons as regards the treatment of personal data and the free circulation of data, effective as of May 25, 2018, and to Organic Law 3/2018, of December 5, on the Protection of Personal Data and guarantee of digital rights.

## Discussion

As far as we know, this is the first randomized clinical trial that examines the effectiveness of an early care therapeutic education and psychological intervention for the management of post-intensive care syndrome and chronic pain after COVID-19.

Patients who survive a critical illness often experience disturbances in many areas, with pain being one of the most important topics. Unfortunately, lack of adequate care to manage this pain is often a problem during the patients stay in the units [5]. This study would quantify the impairment on quality life after ICU admission, as well as the incidence of chronic pain, anxiety, depression, and PTSD. The health status derived from our analysis could be used in economic evaluations of healthcare systems and long-term impact and consequences of the pandemic. Likewise, if the effectiveness of our intervention is verified, it would serve as proof of the need to implement early care programs that allow the recovery process to be followed from early stages, given the current scenario of the pandemic.

Being an integral part of this syndrome, and in order to avoid the limitations and effects of pain on the patient’s quality of life, pain management should be introduced in the ICU.

This project has some limitations.

The main one is that patients meeting inclusion criteria (especially those who have had a more severe course of the disease) may have a severe mobility limitations that would prevent them from attending some trial visits, which would result in a selection bias. This would add to the problem of a small population sample as a consequence of the study being carried out in a single center.

In addition, there are risk factors associated with the development of PICS and chronic pain after a critical illness. These include older age, low socioeconomic status, female gender, previous mental health problems, negative ICU experiences, and delirium.

Therefore, an exploratory analysis of risk factors potentially associated with PICS due to COVID-19 will be carried out, although the sample size will be a determining factor in the conclusions

Future development of multi-center projects may overcome this limitation.

Finally, since this intervention program involves face-to-face visits, the pandemic could limit its correct development.

Restrictions on mobility might make hospital follow-up visits difficult.

## Trial status

Protocol version number 1.0 (April 29, 2020)

Recruitment status: recruiting ongoing (started: May 27, 2020). Recruitment is anticipated to be completed by September 25, 2020, and study completion by March 25, 2021.

## Supplementary Information

**Additional file 1.**

## Abbreviations

PICS: Post-intensive care syndrome
ICU: Intensive care unit
HRQoL: Health-related quality of life
ARDS: Acute respiratory distress syndrome
ICU-AW: Intensive care unit-acquired weakness
MMSE: Mini-mental state exam
VAS: Visual analog scale
EQoL 5D/5 L: European quality of life 5 dimensions/5 levels
BPI: Brief Pain Inventory
HAD: Hospital Anxiety and Depression HAD Scale
