# Supplementary material for: Rationale and study design of an early care, therapeutic education, and psychological intervention program for the management of post-intensive care syndrome and chronic pain after COVID-19 infection (PAIN-COVID): study protocol for a randomized controlled trial
Source: Trials. 2021 Jul 24;22:486. doi: 10.1186/s13063-021-05463-7 (PMC8310406; doi:10.1186/s13063-021-05463-7)
Supplement: Supplementary file 1 — Additional file 1. [file 13063_2021_5463_MOESM1_ESM.docx]

**DEFINITIONS**

**D1: ICU acquired weakness.** Patients who cannot walk with assistance or show significant weakness at ICU discharge (1)

**D2: Central Nervous System degenerative diseases.** Examples: Alzheimer's disease, amyotrophic lateral sclerosis, Lewy body dementia, Parkinson's disease, among others,

**D3: Delirium during ICU admission.** Is defined as a disturbance in attention and awareness that develops over a short period, fluctuates, and is accompanied by a change in cognition, a manifestation of acute brain dysfunction defined by the Diagnostic and Statistical Manual of Mental Disorders (DSM).(2)

**D4: Terminal illness.** Definition according to the palliative care guide, Spanish Society for Palliative Care: “Advanced, progressive, and incurable disease with a lack of reasonable possibilities of specific treatment, with a life prognosis of fewer than 6 months.”(3)

**D5: Sepsis**: should be defined as life-threatening organ dysfunction caused by a dysregulated host response to infection. For clinical operationalization, organ dysfunction can be represented by an increase in the Sequential [Sepsis-related] Organ Failure Assessment (SOFA) score of 2 points or more, which is associated with an in-hospital mortality greater than 10%.(4)

**D6.** **Acute Kidney Injury** In 2012, the Kidney Disease Improving Global Outcomes (KDIGO) defined as absolute or relative increases in SCr and further progressive extent of oliguria, KDIGO describe AKI as a condition that comprise of one or more of the following: (1) an increase in SCr level ≥0.3 mg/dL (≥26.5 µmol/L) within 48 h, or (2) an increase in SCr level to ≥1.5 times baseline, which is known or presumed to have occurred within the prior 7 days, or (3) a urine volume of less than <0.5 mL/kg/h for 6 h or longer. (5)

**D7 stress hyperglycaemia**: fasting glucose >6·9 mmol/L or random glucose >11·1 mmol/L without evidence of previous diabetes), and pre-existing diabetes with deterioration of preillness glycaemic control.

(6)

**D8 Hypoglycemia**: defined as a glucose measurement <3.9 mmol/l. (7)

**QUESTIONNAIRES**


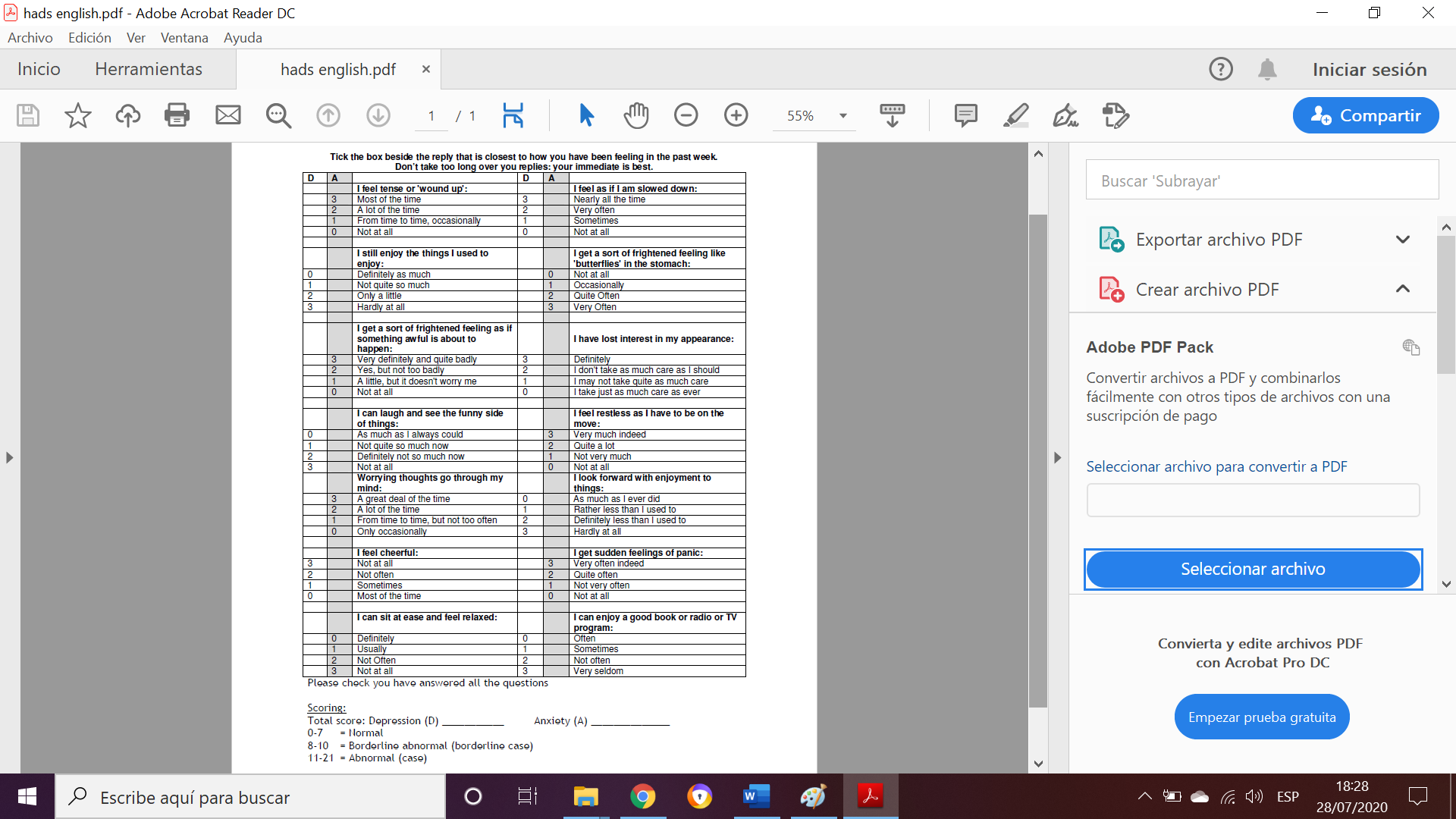
**Q1: Anxiety and Depression scale in hospitalized patients. HAD test. (8)**

**Q1: Escala Ansiedad y Depresión en paciente hospitalarios. Test HAD.(8)**

Este cuestionario trata de saber cómo se encuentra usted. Lea cada frase y marque la respuesta que más se ajusta a cómo se sintió usted durante la semana pasada. No piense mucho las respuestas. Lo más seguro es que si contesta deprisa, sus respuestas reflejen mejor cómo se encontraba usted durante la semana pasada.

| **A1. Me siento tenso/a o nervioso/a:**  3. Casi todo el día  2. Gran parte del día  1. De vez en cuando  0. Nunca | **D2. Todavía disfruto con lo que antes me gustaba:**  0. Como siempre  1. No tanto como antes  2. Solamente un poco  3. Ya no disfruto con nada |
| --- | --- |
| **A3. Siento una especie de temor como si algo malo fuera a suceder:**  3. Sí, y muy intenso  2. Sí, pero no muy intenso  1. Sí, pero no me preocupa  0. No siento nada de eso | **D4. Soy capaz de reírme y ver el lado gracioso de las cosas:**  0. Igual que siempre  1. Actualmente, algo menos  2. Actualmente, mucho menos  3. Actualmente, en absoluto |
| **A5. Tengo la cabeza llena de preocupaciones:**  3. Casi todo el día  2. Gran parte del día  1. De vez en cuando  0. Nunca | **D6. Me siento alegre:**  3. Nunca  2. Muy pocas veces  1. En algunas ocasiones  0. Gran parte del día |
| **A7. Puedo permanecer sentado/a tranquilo/a y relajado/a:**  0. Siempre  1. A menudo  2. Raras veces  3. Nunca | **D8. Me siento lento/a y torpe:**  3. Gran parte del día  2. A menudo  1. A veces  0. Nunca |
| **A9. Experimento una sensación de «nervios y hormigueos» en el estómago:**  0. Nunca  1. Sólo en algunas ocasiones  2. A menudo  3. Muy a menudo | **D10. He perdido el interés por mi aspecto personal:**  3. Completamente  2. No me cuido como debería hacerlo  1. Es posible que no me cuide como debiera  0. Me cuido como siempre lo he hecho |
| **A11. Me siento inquieto/a como si no pudiera parar de moverme:**  3. Realmente mucho  2. Bastante  1. No mucho  0. En absoluto | **D12. Me siento optimista respecto al porvenir:**  0. Como siempre  1. Algo menos que antes  2. Mucho menos que antes  3. En absoluto |
| **A13. Experimento de repente sensaciones de gran angustia o temor:**  3. Muy a menudo  2. Con cierta frecuencia  1. Raramente  0. Nunca | **D14. Me divierto con un buen libro, la radio o un buen programa de radio o TV:**  0. A menudo  1. Algunas veces  2. Pocas veces  3. Casi nunca |
| **Total:** | **Total:** |

**Q2: Quality of life questionnaire. EQ 5D/5L. United Kingdom version. (9)**

**
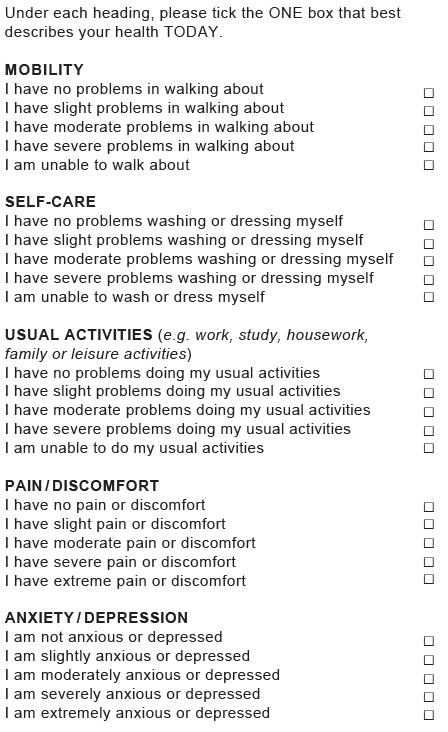
**


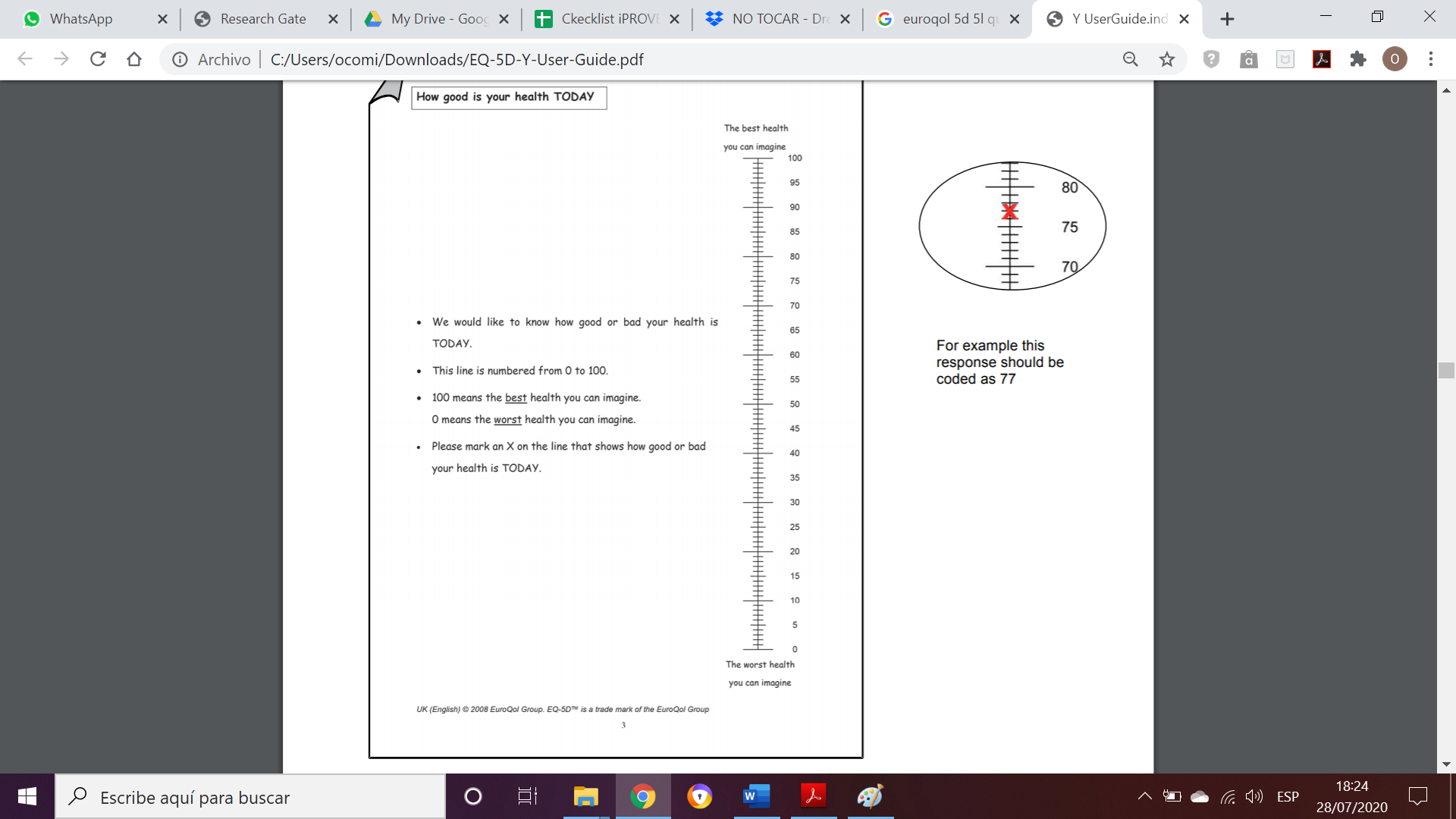


**Q2: Cuestionario de calidad de vida. EQ 5D/5L (9)**

| Debajo de cada enunciado, marque UNA casilla, la que mejor describe su salud HOY. | |
| --- | --- |
| MOVILIDAD |  |
| No tengo problemas para caminar | ❑ |
| Tengo problemas leves para caminar | ❑ |
| Tengo problemas moderados para caminar | ❑ |
| Tengo problemas graves para caminar | ❑ |
| No puedo caminar | ❑ |
| AUTO-CUIDADO |  |
| No tengo problemas para lavarme o vestirme | ❑ |
| Tengo problemas leves para lavarme o vestirme | ❑ |
| Tengo problemas moderados para lavarme o vestirme | ❑ |
| Tengo problemas graves para lavarme o vestirme | ❑ |
| No puedo lavarme o vestirme | ❑ |
| ACTIVIDADES COTIDIANAS *(Ej.: trabajar, estudiar, hacer las tareas domésticas, actividades familiares o actividades durante el tiempo libre)* |  |
| No tengo problemas para realizar mis actividades cotidianas | ❑ |
| Tengo problemas leves para realizar mis actividades cotidianas | ❑ |
| Tengo problemas moderados para realizar mis actividades cotidianas | ❑ |
| Tengo problemas graves para realizar mis actividades cotidianas | ❑ |
| No puedo realizar mis actividades cotidianas | ❑ |
| DOLOR / MALESTAR |  |
| No tengo dolor ni malestar | ❑ |
| Tengo dolor o malestar leve | ❑ |
| Tengo dolor o malestar moderado | ❑ |
| Tengo dolor o malestar fuerte | ❑ |
| Tengo dolor o malestar extremo | ❑ |
| ANSIEDAD / DEPRESIÓN |  |
| No estoy ansioso ni deprimido | ❑ |
| Estoy levemente ansioso o deprimido | ❑ |
| Estoy moderadamente ansioso o deprimido | ❑ |
| Estoy muy ansioso o deprimido | ❑ |
| Estoy extremadamente ansioso o deprimido | ❑ |

**
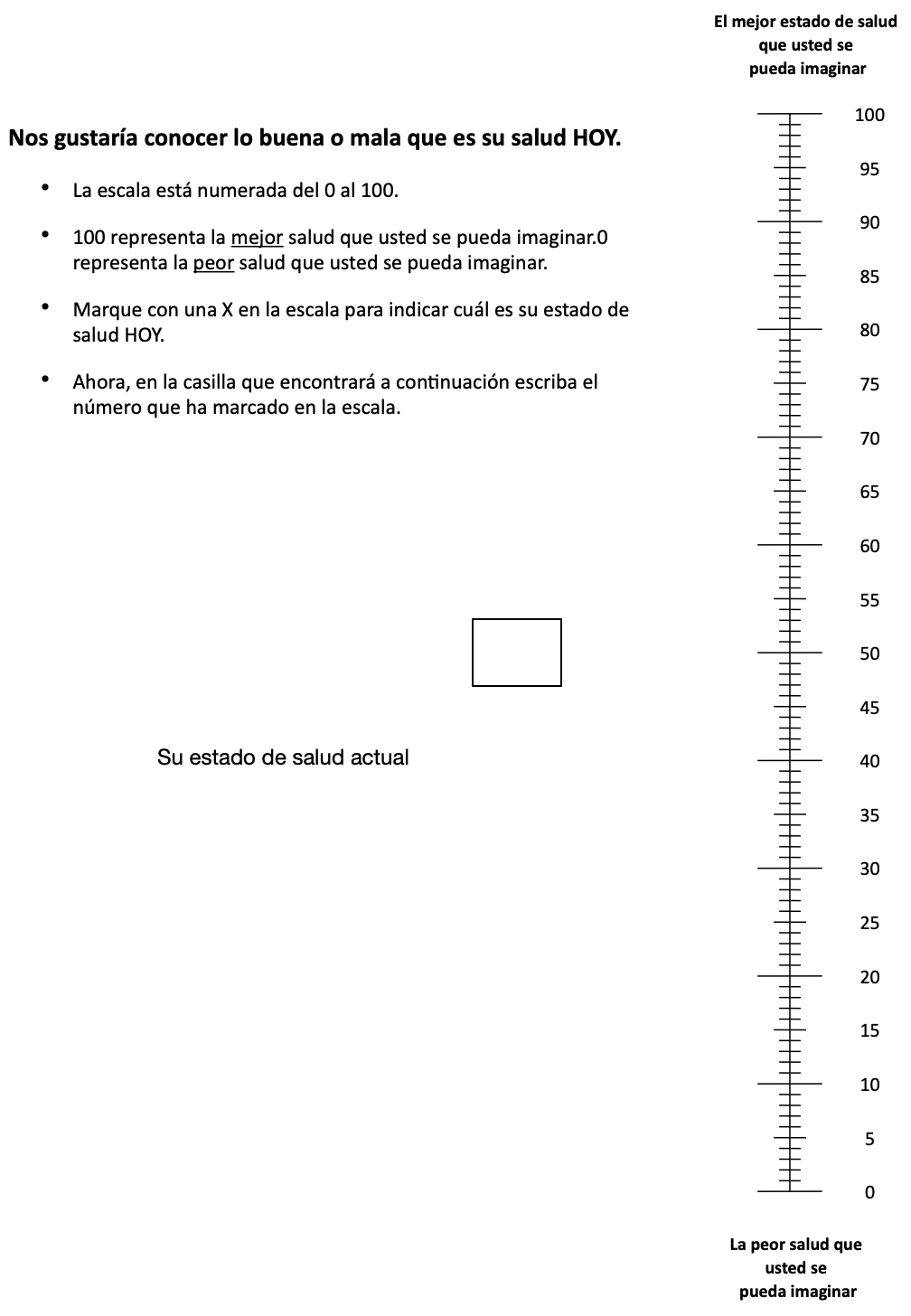
**


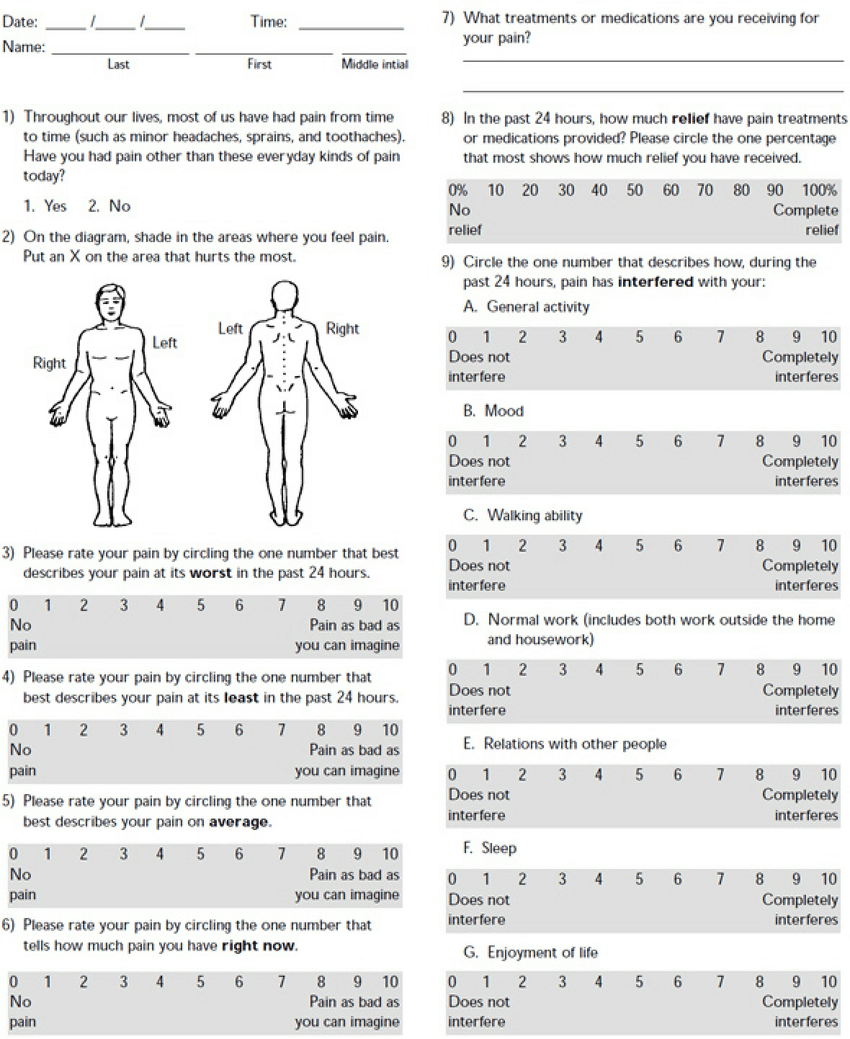
**Q3: Brief Pain Inventory (BPI). Short version.** (10)

**
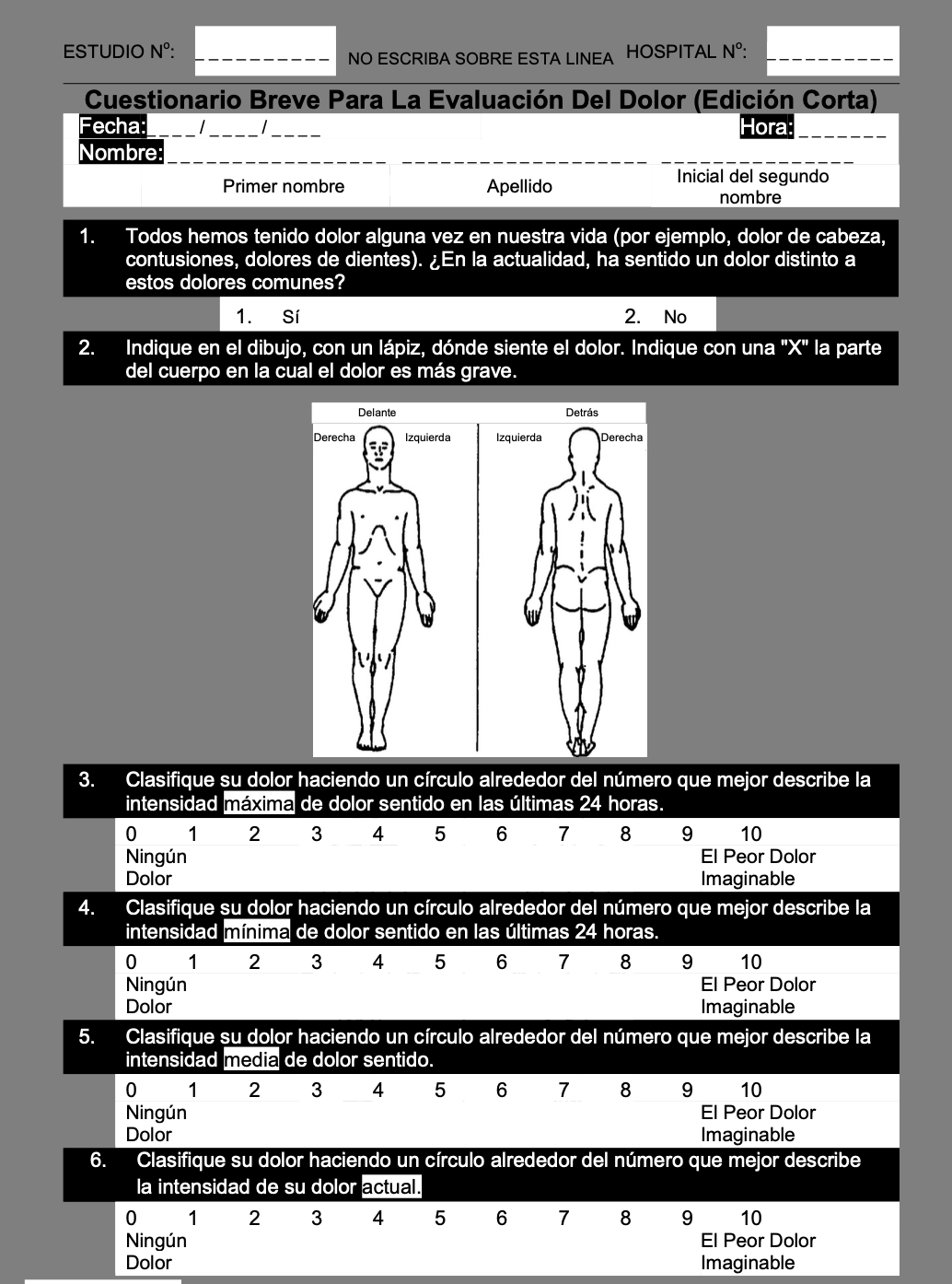
Q3: Cuestionario Breve del Dolor (CBD). Versión Corta.** (8)

**
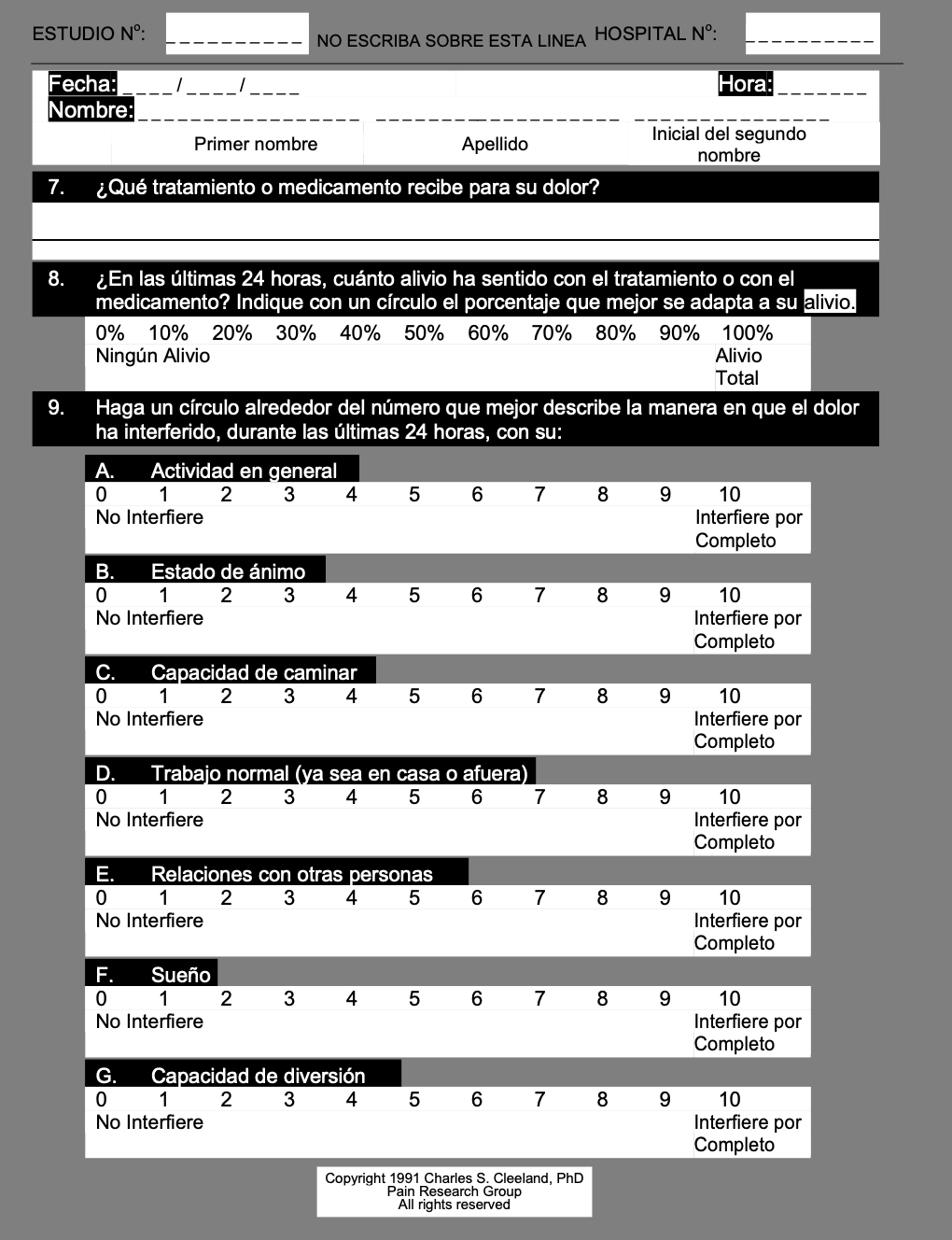
**

**Q4: Pain Catastrophizing Scale. (11)**

Everyone experiences painful situations at some point in their lives. Such experiences may include headaches, tooth pain, joint or muscle pain. People are often exposed to situations that may cause pain such as illness, injury, dental procedures or surgery.

We are interested in the types of thoughts and feeling that you have when you are in pain. Listed below are thirteen statements describing different thoughts and feelings that may be associated with pain. Using the scale, please indicate the degree to which you have these thoughts and feelings when you are experiencing pain.

|  | *Not at all* | *To a slight degree* | *To a moderate degree* | To a great degree | All the time |
| --- | --- | --- | --- | --- | --- |
| 1. I worry all the time about whether the pain will end | 0 | 1 | 2 | 3 | 4 |
| 2. I feel I can’t go on | 0 | 1 | 2 | 3 | 4 |
| 3. It’s terrible and I think it’s never going to get any better | 0 | 1 | 2 | 3 | 4 |
| 4. It’s awful and I feel that it overwhelms me | 0 | 1 | 2 | 3 | 4 |
| 5. I feel I can’t stand it anymore | 0 | 1 | 2 | 3 | 4 |
| 6. I become afraid that the pain will get worse | 0 | 1 | 2 | 3 | 4 |
| 7. I keep thinking of other painful events | 0 | 1 | 2 | 3 | 4 |
| 8.I anxiously want the pain to go away | 0 | 1 | 2 | 3 | 4 |
| 9. I can’t seem to keep it out of my mind | 0 | 1 | 2 | 3 | 4 |
| 10. I keep thinking about how much it hurts | 0 | 1 | 2 | 3 | 4 |
| 11. I keep thinking about how badly I want the pain to stop | 0 | 1 | 2 | 3 | 4 |
| 12. There’s nothing I can do to reduce the intensity of the pain | 0 | 1 | 2 | 3 | 4 |
| 13. I wonder whether something serious may happen | 0 | 1 | 2 | 3 | 4 |

**Q4: Escala de catastrofización ante el dolor. (11)**

Todas las personas experimentamos situaciones de dolor en algún momento de nuestra vida. Tales experiencias pueden incluir dolor de cabeza, dolor de muelas, dolor muscular o de articulaciones. Las personas estamos a menudo expuestas a situaciones que pueden causar dolor como las enfermedades, las heridas, los tratamientos dentales o las intervenciones quirúrgicas. Este cuestionario nos puede servir para conocer el tipo de pensamientos y sentimientos que usted tiene cuando siente dolor.

A continuación, se presenta una lista de 13 frases que describen diferentes pensamientos y

sentimientos que pueden estar asociados al dolor. Utilizando la siguiente escala, por favor, indique el grado en que usted tiene esos pensamientos y sentimientos cuando siente dolor.

|  | *Nada en Absoluto* | *Un poco* | *Moderadamente* | Mucho | Todo el tiempo |
| --- | --- | --- | --- | --- | --- |
| 1. Estoy preocupado todo el tiempo pensando en si el dolor desaparecerá | 0 | 1 | 2 | 3 | 4 |
| 2. Siento que ya no puedo más | 0 | 1 | 2 | 3 | 4 |
| 3. Es terrible y pienso que esto nunca va a mejorar | 0 | 1 | 2 | 3 | 4 |
| 4. Es horrible y siento que esto es más fuerte que yo | 0 | 1 | 2 | 3 | 4 |
| 5. Siento que no puedo soportarlo más | 0 | 1 | 2 | 3 | 4 |
| 6. Temo que el dolor empeore | 0 | 1 | 2 | 3 | 4 |
| 7. No dejo de pensar en otras situaciones en las que experimento dolor | 0 | 1 | 2 | 3 | 4 |
| 8. Deseo desesperadamente que desaparezca el dolor | 0 | 1 | 2 | 3 | 4 |
| 9. No puedo apartar el dolor de mi mente | 0 | 1 | 2 | 3 | 4 |
| 10. No dejo de pensar en lo mucho que me duele | 0 | 1 | 2 | 3 | 4 |
| 11. No dejo de pensar en lo mucho que deseo que desaparezca el dolor | 0 | 1 | 2 | 3 | 4 |
| 12. No hay nada que pueda hacer para aliviar la intensidad del dolor | 0 | 1 | 2 | 3 | 4 |
| 13. Me pregunto si me puede pasar algo grave | 0 | 1 | 2 | 3 | 4 |

**Q5. DN4 Interview (12,13)**

**To estimate the probability of neuropathic pain, please answer yes or no for each item of the following questions**

**INTERVIEW OF THE PATIENT**

QUESTION 1: Does the pain have one or more of the following characteristics?

|  |  | Yes |  | No |
| --- | --- | --- | --- | --- |
| 1 | Burning |  |  |  |
| 2 | Painful Cold |  |  |  |
| 3 | Electricshocks |  |  |  |

QUESTION 2: Is the pain associated with one or more of the following
symptoms in the same area?

|  |  | Yes |  | No |
| --- | --- | --- | --- | --- |
| 4 | Tingling |  |  |  |
| 5 | Pins and needles |  |  |  |
| 6 | Numbness |  |  |  |
| 7 | Itching. |  |  |  |

YES = 1 point NO = 0 points

**Patient**’**s Score: : / 10**

**Q5: DN4 Entrevista**

Responda a las preguntas siguientes marcando sí o no en la casilla correspondiente.

**ENTREVISTA AL PACIENTE**

Pregunta 1: ¿Tiene su dolor alguna de estas características?

|  |  | Si | No |
| --- | --- | --- | --- |
| 1 | Quemazón |  |  |
| 2 | Sensación de frío doloroso |  |  |
| 3 | Descargas eléctricas |  |  |

Pregunta 2: ¿Tiene en la zona donde le duele alguno de estos síntomas?

|  |  | Si | No |
| --- | --- | --- | --- |
| 4 | Hormigueo |  |  |
| 5 | Pinchazos |  |  |
| 6 | Entumecimiento |  |  |
| 7 | Escozor |  |  |

Puntuación : / 10


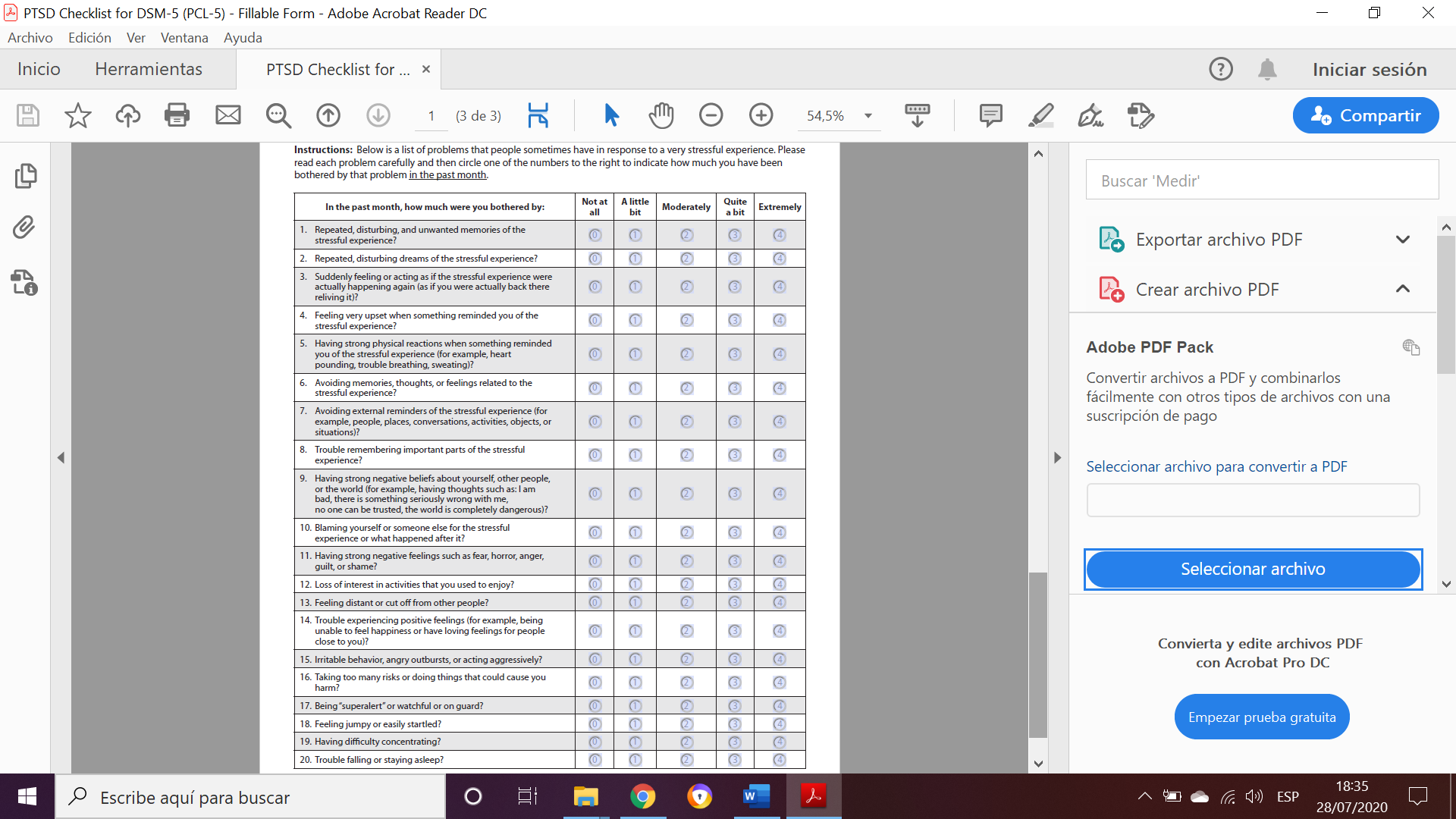
**Q6: PTSD Checklist (PCL-5). English version. (14,15)**

**Q6: PTSD Checklist (PCL-5) . Traducción al Español.** (11)

A continuación hay una lista de problemas que a veces tienen las personas en respuesta a una experiencia muy estresante. Por favor, lea cada problema cuidadosamente y luego señale uno de los números a la derecha para indicar cuánto le molestó ese problema desde que estuvo ingresado en la unidad de cuidados intensivos.

| **Desde que ha estado ingresado en la UCI**  **¿cuanta molestia ha sentido por:** | Nada | Un Poco | Moderadamente | Bastante | Extremadamente |
| --- | --- | --- | --- | --- | --- |
| **1.)** recuerdos repetidos, perturbadores e indeseados sobre la estancia en UCI? | 0 | 1 | 2 | 3 | 4 |
| **2.)** sueños repetidos y perturbadores de su estancia en UCI? | 0 | 1 | 2 | 3 | 4 |
| **3.)** actuar o sentir de repente como si la experiencia de la UCI ocurriera otra vez (como si lo volviera a vivir)? | 0 | 1 | 2 | 3 | 4 |
| **4.)** sentirse muy angustiado cuando algo le recuerda la estancia en la UCI? | 0 | 1 | 2 | 3 | 4 |
| **5.)**. tener reacciones físicas cuando algo le recuerda su estancia en UCI (como latidos fuertes del corazón, le cuesta respirar, suda mucho) ? | 0 | 1 | 2 | 3 | 4 |
| **6.)**  evitar recuerdos, pensamientos o sentimientos relacionados con su estancia en UCI ? | 0 | 1 | 2 | 3 | 4 |
| **7.)** evitar personas, lugares, conversaciones, actividades, objetos o situaciones que le recuerden su estancia en UCI ? | 0 | 1 | 2 | 3 | 4 |
| **8.)**  dificultad para recordar lo que pasó durante su estancia en UCI (sin contar lo que no podría recordar por estar inconsciente)? | 0 | 1 | 2 | 3 | 4 |
| **9.)** tener creencias negativas fuertes acerca de sí mismo, de otras personas o del mundo (por ejemplo, tener pensamientos como: soy malo, hay algo en mí que está muy mal, no se puede confiar en nadie, el mundo es completamente peligroso)? | 0 | 1 | 2 | 3 | 4 |
| **10.)** culparse a sí mismo o a otros por la experiencia durante la UCI, o por lo que sucedió́ después de esta? | 0 | 1 | 2 | 3 | 4 |
| **11.)**  tener sentimientos negativos intensos, como miedo, terror, ira, culpa o vergüenza? | 0 | 1 | 2 | 3 | 4 |
| **12.)**  perdida de interés en actividades que antes disfrutaba? | 0 | 1 | 2 | 3 | 4 |
| **13.)**  sentirse distante o enajenado de otras personas? | 0 | 1 | 2 | 3 | 4 |
| **14)** dificultad para sentir emociones positivas (por ejemplo, ser incapaz de sentir alegría o sentimientos de amor por las personas cercanas a usted)? | 0 | 1 | 2 | 3 | 4 |
| **15.)** irritabilidad, explosiones de rabia o actuar agresivamente? | 0 | 1 | 2 | 3 | 4 |
| **16.)** tomar demasiados riesgos o hacer cosas que pudieron haberle causado daño? | 0 | 1 | 2 | 3 | 4 |
| **17.)** estar "extremadamente alerta", o vigilante, o en guardia? | 0 | 1 | 2 | 3 | 4 |
| **18.)** sentirse muy nervioso o sobresaltarse fácilmente? | 0 | 1 | 2 | 3 | 4 |
| **19.)** tener dificultad para concentrarse? | 0 | 1 | 2 | 3 | 4 |
| **20.)** tener dificultad para dormirse o mantener el sueño ? | 0 | 1 | 2 | 3 | 4 |

**TABLES**

**Table 1. Psychological Interventions.**

|  |  |
| --- | --- |
| *SESSION OBJECTIVES* | ACTIVITIES: |
| ***Session 1:***  *To establish a good therapeutic relationship and introduce and explain the principles of psychological treatment. Information will be given on what depression is and the factors that maintain it.* | **a.** Presentation by therapists and group members.  **b.** Instructions for making the records. Delivery of self-observation record sheets.  **c.** Making a list of positive activities.  **d.** Relaxation training (progressive muscle relaxation and training in  diaphragmatic breathing). |
| ***Session 2:***  *To raise awareness of the relationship between pleasant activities and the improvement of the mood from the patient's self-records.* | **a.** Conducting the Mood Exercise and Activity. Introduction long-term positive effects of behaviors.  **b.** Continue the self -observation log for the following week and note at least one long-term positive effect for one activity. Mark it the less for an activity for each day. |
| ***Session 3 and 4:***  *To establish of realistic goals adapted to the capacities and context of the patient and their segmentation into sub-goals. Teach you to set goals, sub-goals, and make sure that the goals you set depend on your real abilities and not on external factors.* | **a**. Analysis of the patient's decision-making process and planning of activities. Analyze what you do rigidly and unrealistically, with expectations that are too high and unattainable. Importance of modifying goals when necessary.  **b.** Define the goals in an operational way, with sub-goals. Point out positive activities during the week that help you achieve your goals. |
| ***Session 5:***  *To analyze the patient's attributional process of their successes and failures, based on Seligman's attributional model.* | **a**. Exercise on attribution of responsibility. |
| ***Session 6:***  *To acquire a strategy to administer self-reinforcements on a contingent basis.* | **a.** Preparation of two lists  **-** List of positive values: list of positive statements that the patient must make about himself .  **-** Reinforcement menu: list of positive activities easily achievable by the subject |
| ***Session 7:***  *To promote that the clinical improvement of the patients is consolidated and maintained in a stable, consistent and lasting way.* | **a.** The evolution of the problem from the beginning to the end of the treatment is analyzed, the techniques learned throughout the sessions are summarized, the improvement is attributed to both the treatment and the patient, and the coping of possible future problems |
|  |  |

**Table 2. Demographic Data and Patient Characteristics**

| Age | In years |
| --- | --- |
| Sex | Female/Male |
| Body Mass Index | Body weight (Kg) divided by the square of height (m2) |
| Educational Status | None / Primary Schoo Not completed ; Primary School Completed;  Secondary School; Post-Secondary School |
| Current tobacco smoking status | Nº of respondents who currently smoke tobacco daily |
| Psychiatric disease´s history | Diagnosed by a psychiatrist. Dichotmic Variable: Yes/No |
| Type of psquiatric disease | Major depressive disorder, Obsessive-Compulsive disorder, Bipolar disorder, Substance Use disorder  According to DSM V definition.: (V. Hasin DS et al. Am J Psychiatry. 2013 Aug; 170 (8): 834-51). |
| Severe Mental Disorder | Psychotic disorders (excluding organic ones), persisted at least 2 years, or with progressive and significant impairment in the functional status in the last 6 months, although it symptoms remit and cause disability that produces significant functional limitations daily life activities.  (Grupo de Trabajo de la Guía de Práctica Clínica de Intervenciones Psicosociales en el Trastorno Mental Grave. Guía de Práctica Clínica de Intervenciones Psicosociales en el Trastorno Mental Grave. Plan de Calidad para el Sistema Nacional de Salud del Ministerio de Sanidad y Política Social. Instituto Aragonés de Ciencias de la Salud-I+CS; 2009. Guías de Práctica Clínica en el SNS: I+CS No 2007/05). |
| History of chronic pain | More than occasional pain (e.g. short headache/toothache) in the last 4 weeks weeks before ICU admission  Dichotmic Variable: Yes/No  (P. Baumbach et al. Eur J Pain 22 (2018) 402--413) |
| Chronic Pain Type | - Widespread Pain: Pain located axially, above and below the waist, and on both sides of the body (ACR 1990 definition; Wolfe F, et al . Arthritis Care Res 2010;62:600–10) - Localized Pain: pain at a specific site on the body - Cancer Pain: pain related to a cancer disease - Neurophatic Pain: diagnosed by their referring physicians |
| Opioid Tolerance Patient | History of ≥ 60 mg morphine equivalent dose in the 7 days before been admitted to ICU (Edwards et al (Anesth Analg 2019;129:553–66). |
| Previous ICU admission. | Dichotmic Variable: Yes/No |

**REFERENCES**

1. Busico M, das Neves A, Carini F, Pedace M, Villalba D, Foster C, et al. Follow-up program after intensive care unit discharge. Medicina Intensiva (English Edition). 2019 May;43(4):243–54.

2. American Psychiatric Association. Diagnostic and Statistical Manual of Mental Disorders [Internet]. Fifth Edition. American Psychiatric Association; 2013 [cited 2021 Jan 31]. Available from: http://psychiatryonline.org/doi/book/10.1176/appi.books.9780890425596

3. SECPAL [Internet]. [cited 2021 Jan 31]. Available from: https://www.secpal.com/guia-cuidados-paliativos-1

4. Singer M, Deutschman CS, Seymour CW, Shankar-Hari M, Annane D, Bauer M, et al. The Third International Consensus Definitions for Sepsis and Septic Shock (Sepsis-3). JAMA. 2016 Feb 23;315(8):801.

5. Kidney Disease: Improving Global, Outcomes (KDIGO) Acute Kidney Injury Work Group. KDIGO Clinical Practice Guideline for Acute Kidney Injury. Kidney Int Suppl (2011). 2012;2(1):8–12.

6. Dungan KM, Braithwaite SS, Preiser J-C. Stress hyperglycaemia. Lancet. 2009 May 23;373(9677):1798–807.

7. Workgroup on Hypoglycemia, American Diabetes Association. Defining and reporting hypoglycemia in diabetes: a report from the American Diabetes Association Workgroup on Hypoglycemia. Diabetes Care. 2005 May;28(5):1245–9.

8. Herrero MJ, Blanch J, Peri JM, De Pablo J, Pintor L, Bulbena A. A validation study of the hospital anxiety and depression scale (HADS) in a Spanish population. Gen Hosp Psychiatry. 2003 Aug;25(4):277–83.

9. Janssen MF, Pickard AS, Golicki D, Gudex C, Niewada M, Scalone L, et al. Measurement properties of the EQ-5D-5L compared to the EQ-5D-3L across eight patient groups: a multi-country study. Qual Life Res. 2013 Sep;22(7):1717–27.

10. Brief Pain Inventory (BPI) [Internet]. MD Anderson Cancer Center. [cited 2021 Jan 31]. Available from: https://www.mdanderson.org/research/departments-labs-institutes/departments-divisions/symptom-research/symptom-assessment-tools/brief-pain-inventory.html

11. Darnall BD, Sturgeon JA, Cook KF, Taub CJ, Roy A, Burns JW, et al. Development and Validation of a Daily Pain Catastrophizing Scale. J Pain. 2017 Sep;18(9):1139–49.

12. Haanpää M, Attal N, Backonja M, Baron R, Bennett M, Bouhassira D, et al. NeuPSIG guidelines on neuropathic pain assessment. Pain. 2011 Jan;152(1):14–27.

13. Bouhassira D, Attal N, Alchaar H, Boureau F, Brochet B, Bruxelle J, et al. Comparison of pain syndromes associated with nervous or somatic lesions and development of a new neuropathic pain diagnostic questionnaire (DN4). Pain. 2005 Mar;114(1–2):29–36.

14. Weathers, F.W., Litz, B.T., Keane, T.M., Palmieri, P.A., Marx, B.P., & Schnurr, P.P. The PTSD Checklist for DSM-5 (PCL-5). [Internet]. 2013. Available from: Scale available from the National Center for PTSD at www.ptsd.va.gov.

15. Blevins CA, Weathers FW, Davis MT, Witte TK, Domino JL. The Posttraumatic Stress Disorder Checklist for DSM-5 (PCL-5): Development and Initial Psychometric Evaluation. J Trauma Stress. 2015 Dec;28(6):489–98.
